# Latin America’s Dengue Outbreak Poses a Global Health Threat

**DOI:** 10.3390/v17010057

**Published:** 2025-01-01

**Authors:** Michelle Teixeira de Almeida, Davi Gabriel Salustiano Merighi, Aline Biazola Visnardi, Cauê Augusto Boneto Gonçalves, Vitor Martins de Freitas Amorim, Anielle Salviano de Almeida Ferrari, Anacleto Silva de Souza, Cristiane Rodrigues Guzzo

**Affiliations:** Department of Microbiology, Institute of Biomedical Sciences, University of São Paulo, São Paulo 5508-900, Brazil; michelle.almeida@usp.br (M.T.d.A.); davism@usp.br (D.G.S.M.); caue.boneto@usp.br (C.A.B.G.); anielle.ferrari@usp.br (A.S.d.A.F.)

**Keywords:** Dengue, outbreak, vaccines, pandemic, epidemiology

## Abstract

Dengue fever, caused by the dengue virus (DENV), poses a significant global health challenge, particularly in tropical and subtropical regions. Recent increases in indigenous DENV cases in Europe are concerning, reflecting rising incidence linked to climate change and the spread of *Aedes albopictus* mosquitoes. These vectors thrive under environmental conditions like temperature and humidity, which are increasingly influenced by climate change. Additionally, global travel accelerates the cross-border spread of mosquito-borne diseases. DENV manifests clinically in a spectrum from asymptomatic cases to severe conditions like dengue hemorrhagic fever and dengue shock syndrome, influenced by viral serotype and host factors. In 2024, Brazil experienced a fourfold increase in dengue cases compared to 2023, accompanied by higher mortality. Conventional control measures, such as vector control, community engagement, and vaccination, proved insufficient as climate change exacerbated mosquito proliferation, challenging containment efforts. In this regard, our review analyzes prevention measures and therapeutic protocols during the outbreak while addressing DENV transmission dynamics, clinical presentations, and epidemiological shifts. It also evaluates diagnostic strategies combining clinical assessment with serological and molecular testing, providing information to improve diagnostic and preventive measures. The global expansion of dengue-endemic regions, including outbreaks in Europe, highlights the urgent need for enhanced surveillance, proactive interventions, and international collaboration to mitigate the growing threat of Dengue and other arboviruses like West Nile, Zika, Chikungunya, Oropouche, and Yellow Fever viruses.

## 1. Introduction

Dengue is a viral zoonotic disease transmitted by arthropod vectors, primarily mosquitoes of the *Aedes* genus (*Ae.*), notably *Ae. aegypti* and *Ae. albopictus* (Diptera, Culicidae) [1]. Over the past three decades, the incidence of arboviral diseases has increased markedly, driven by factors such as climate change, population growth, and global travel, which collectively create favorable conditions for viral transmission [2]. Extreme weather events, including heat waves and floods, have contributed to the geographic expansion of mosquito populations, facilitating the spread of arboviruses such as Dengue, Chikungunya, Yellow Fever, Rift Valley Fever, West Nile Fever, Japanese Encephalitis, and Zika [2,3]. Global warming exacerbates these trends, enabling the migration of mosquito vectors to previously unaffected regions across Africa, Asia, Europe, and the Americas [4,5,6]. Arboviruses follow a complex transmission cycle involving arthropod vectors and vertebrate hosts [7], with the human–mosquito cycle predominantly occurring in urban environments [8]. Viral transmission from humans to mosquitoes is influenced by the host’s viral load; individuals nearing the peak of viremia are more likely to infect mosquitoes during feeding, increasing the probability of subsequent viral transmission [8,9].

In addition to mosquitoes, other vectors, such as ticks, sandflies, and biting midges, also play significant roles in transmitting various arboviruses [10,11]. The families of viruses that encompass arboviruses include: Flaviviridae (genus *Flavivirus*), Bunyaviridae (genera *Nairovirus*, *Orthobunyavirus*, *Phlebovirus*, and *Tospovirus*), Togaviridae (genus *Alphavirus*), Rhabdoviridae (genus *Vesiculovirus*), Orthomyxoviridae (genus *Thogotovirus*), and Reoviridae (genera *Orbivirus* and *Coltivirus*) [12,13]. Among these, the most significant viruses causing diseases in humans and animals belong to the *Togaviridae* and *Flaviviridae* families [12,14]. Flaviviruses are a group of enveloped, (+)ssRNA viruses [15,16] that includes Dengue Virus (DENV), Japanese Encephalitis Virus (JEV), West Nile Virus (WNV), Zika Virus (ZIKV), Yellow Fever Virus (YFV), and Tick-Borne Encephalitis Virus (TBEV) [15]. Other viruses present in the Americas are Chikungunya Virus (CHIKV) and Mayaro Virus (MAYV) that have (+)ssRNA, which belong to the *Alphavirus* genus of the *Togaviridae* family [17,18]. Another virus of concern is the Oropouche Virus (OROV), a segmented (-)ssRNA virus belonging to the genus *Orthobunyavirus* in the *Peribunyaviridae* family, which causes symptoms similar to dengue fever [18,19].

Dengue is an endemic disease affecting over 100 countries, with the highest prevalence in the tropical and subtropical regions of Southeast Asia, Africa, the Western Pacific, and the Americas [20,21]. These regions provide favorable temperature and humidity conditions for the proliferation of mosquito vectors [22,23]. Cases have also been reported in parts of Europe and the United States, indicating the disease’s expanding geographic range [21]. Climate change, population growth, and increased global travel are anticipated to further elevate dengue incidence, particularly in endemic regions. The ongoing spread of *Aedes* mosquito vectors, originally from Asia, is projected to push the virus into new territories, including regions previously unaffected by dengue [9,24,25]. In Brazil, dengue epidemics are closely associated with temperature fluctuations and rainfall patterns. Mosquito populations increase during warm, wet periods, which provide optimal conditions for their reproduction. Consequently, most cases of dengue fever occur following humid and hot seasons, particularly in densely populated urban areas. High temperatures (26 °C to 29 °C) significantly impact the spreading of dengue by accelerating mosquito development, enhancing reproduction and survival rates, increasing biting frequency, and promoting viral replication within the vector [26].

The DENV has four distinct serotypes, each exhibiting genetic diversity and displaying different interactions with the antibodies in human blood serum [27]. These serotypes are classified into various genotypes based on samples collected from different geographic regions: (1) DENV-1 includes I, II, III, and IV genotypes; (2) DENV-2 is categorized into Asian genotype I, Asian genotype II, Cosmopolitan genotype, American genotype V, and Sylvatic genotype; (3) DENV-3 comprises I, II, III, and IV genotypes; (4) DENV-4 encompasses I, II, III, and IV genotypes [28]. Recently, a fifth serotype, DENV-5, was identified in Malaysia [29,30]. Human infection occurs through the bite of a female mosquito, particularly *Ae. aegypti* [31,32] or *Ae. albopictus* [33], and affects approximately 390 million people annually. While *Ae. aegypti* is commonly associated with most infections, the geographic range of *Ae. albopictus* continues to expand, driven by its ecological plasticity, which allows it to tolerate colder climates. It is competitive feeding behavior and the lack of effective control strategies further contribute to the increasing number of dengue cases in previously colder regions [8,34,35].

**Figure 1:**
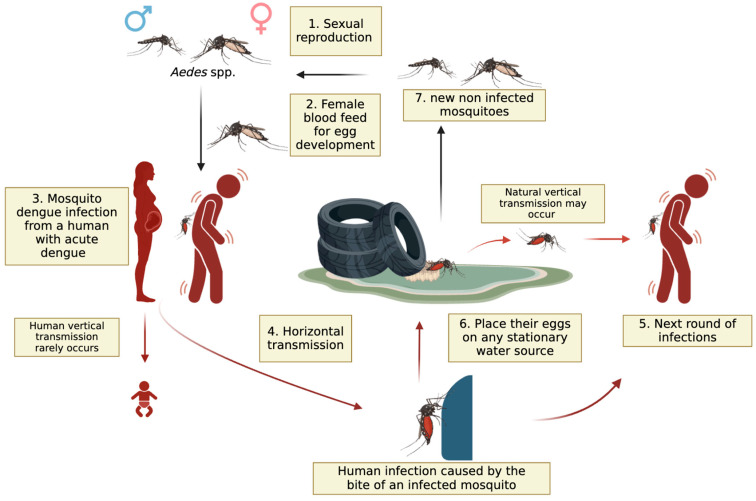
Reproduction cycle of *Aedes* spp. and dengue transmission cycle. The reproduction cycle of *Aedes* spp. mosquitoes and the dengue transmission cycle involve several stages. Male and female mosquitoes mate (step 1), and female mosquitoes go for blood feeding for egg development (step 2). A female mosquito then bites an infected individual, acquiring the Dengue virus (step 3 and 4). This infected mosquito subsequently bites multiple healthy individuals, transmitting the virus to them (step 5). These newly infected individuals then transmit the virus to additional mosquitoes that bite them. The female mosquito lays her eggs in stagnant water sources (step 6), where the eggs hatch into larvae and develop into new mosquitoes, perpetuating the cycle (step 7). Two types of vertical DENV transmission may occur: from an infected pregnant woman to her baby, which is rare [41], and natural vertical transmission of DENV in *Ae. aegypti* and *Ae. albopictus* mosquito populations, which serves as a mechanism for viral persistence in the environment during periods unfavorable for horizontal transmission [22,43,44,45]. This last process is an important maintenance strategy for DENV circulation, ensuring the virus remains within mosquito populations even when conditions limit transmission between mosquitoes and human hosts. The figure was designed with BioRender.

When a person infected with DENV is bitten by an *Ae. aegypti* or *Ae. albopictus*, a complex sequence of events allows the virus to replicate and spread within the mosquito [33,36] (Figure 1). Inside the female *Aedes* mosquito, the virus infects the midgut epithelial cells, where it binds to specific receptors and replicates [37]. After replication, the virus exits the midgut and spreads throughout the hemocoel, infecting various secondary tissues, such as the fat body, nervous system, and salivary glands. Infection of the salivary glands is essential for the virus’s transmission to new hosts [38,39,40]. When the mosquito bites another person, DENV is transmitted through the saliva injected during blood feeding, introducing the virus into the new host’s bloodstream and initiating a new cycle of infection [38]. This entire process, from the ingestion of the virus to the mosquito’s ability to transmit it to a new host, typically takes around 8–12 days, depending on environmental factors such as temperature and humidity. Given Brazil’s tropical climate, where dengue is a concern, these environmental factors are especially relevant, as warmer temperatures can shorten the time needed for the virus to become transmissible [36,38]. This period is known as the extrinsic incubation period [22]. Understanding these steps is crucial for developing strategies to interrupt the transmission cycle of DENV and reduce the incidence of dengue worldwide. Vertical transmission from human to human in the case of dengue from mother to child has been reported, although there is no evidence of such transmission in early pregnancy. It can occur in late pregnancy, with studies showing an increased risk of low birth weight and preterm births among infected pregnant women. However, the significance of vertical transmission as a risk factor for adverse pregnancy outcomes remains inconclusive [41], and congenital dengue infection is regarded as rare in the literature [42]. Notably, natural vertical transmission in *Ae. aegypti* and *Ae. albopictus* is regarded as a maintenance mechanism for the DENV during unfavorable conditions, potentially contributing to the emergence of dengue outbreaks. The detection of DENV-3 during years when no human autochthonous cases of this serotype were recorded suggests the silent circulation of DENV-3, indicating that green areas may sustain serotypes not currently circulating in the human population, possibly through vertical transmission mechanisms [43].

Once inside the human host, the Dengue virus infects a range of cells and tissues, such as macrophages, lymphocytes, and endothelial cells, as well as organs like the lymph nodes, lungs, liver, kidneys, stomach, and the central nervous system. These diverse targets contribute to the virus’s systemic impact throughout the body, as described in more detail below [46]. The dengue infection progresses through three phases: the febrile phase, the critical phase, and the recovery phase [47]. The febrile phase lasts about a week, characterized by high fever, severe headache, muscle and joint pain, eye pain, fatigue, nausea, vomiting, loss of appetite, and minor bleeding [48,49]. The critical phase involves more severe symptoms, such as plasma leakage and internal hemorrhage [7]. The recovery phase is marked by the gradual reabsorption of fluid from the extravascular compartment, improvement in general well-being, return of appetite, and stabilization of the hemodynamic state [7]. Additionally, neurological complications such as encephalopathy, myelopathy, myositis, and peripheral neuropathy are frequently identified following dengue infection [50].

This review reports dengue fever as an escalating global health challenge, with a particular focus on Latin America, where environmental factors, rapid urbanization, and socio-economic conditions create an ideal environment for the *Ae. aegypti* mosquito. In 2024, reported cases in Brazil surged more than fourfold compared to 2023. Climate variability and unequal access to healthcare exacerbate the situation. The study also explores dengue transmission, influenced by climatic factors, and its clinical manifestations, ranging from asymptomatic to severe forms such as hemorrhagic fever and shock syndrome. Prevention strategies include vector control, community engagement, and vaccination campaigns, but face challenges such as insecticide resistance and the difficulty of implementing certain measures in tropical climates. The spreading of dengue to new regions, including Europe, underscores the urgent need for global collaboration to mitigate the disease.

## 2. The Impact of Global Warming on Dengue Outbreaks in the Latin America

As global warming becomes more evident with the increasing frequency of climate disasters, one significant impact is the intensification of rainfall [51]. The Brazilian National Institute for Space Research (INPE) reported that in 2024, the southern part of Brazil will experience up to a 30% increase in average annual rainfall compared to the last three decades [52]. This increase in rainfall will lead to more standing water in urban areas, which are the primary habitats for the main vectors of Dengue [53]. The proliferation of *Aedes* mosquitoes is directly related to precipitation, humidity, and high temperatures, with Latin America providing an ideal environment. Global warming leads to higher temperatures in subtropical and temperate areas, which often have high urban population densities, creating ideal conditions for proliferation of mosquitoes. These observations suggest that global warming and climate change are directly influencing the spread of tropical diseases. In developing countries, which are experiencing an increase in natural disasters such as floods, tsunamis, and storms, these events exacerbate the formation of standing water in large urban areas. This, in turn, facilitates the spread of dengue and other mosquito-borne diseases.

**Figure 2:**
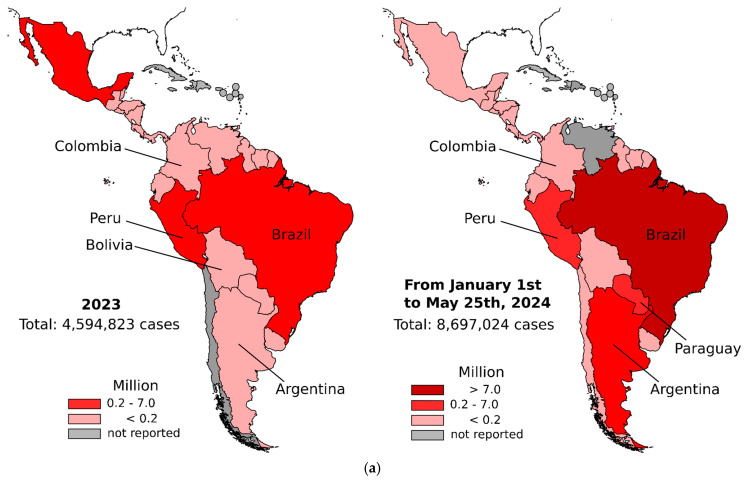

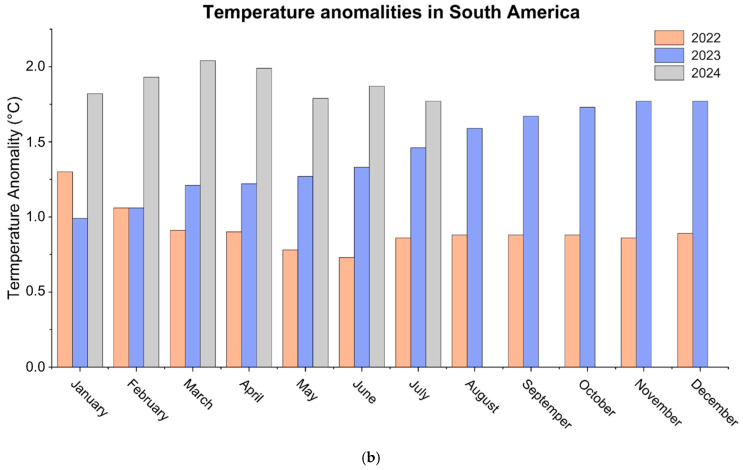
Distribution of the total number of dengue cases, including both confirmed cases and cases under investigation, in Latin American countries (2023–2024) and average temperature anomalies (2022–2024). (**a**) Dengue cases in Latin America exhibited significant regional variation in 2023 and from January to May 2024, with marked increases in several countries. Brazil presented a largest proportion of cases, with a notable escalation from 3,064,739 cases in 2023 to 7,253,599 cases until May of 2024. Similarly, Argentina reported an increase of dengue cases from 146,876 in 2023 to 498,091 in 2024, while Paraguay experienced an increase from 63,216 to 278,827. Nicaragua showed substantial decrease in case numbers. This figure was performed using data obtained from the Pan American Health Organization (PAHO). Graph colored based on the dengue fever cases shown in Table 1. (**b**) Temperature anomalies in South America in the period of 2022–2024.

The data on dengue cases in Latin America show significant variations between 2023 and 2024 (Figure 2 and Table 1). Brazil leads with an increase in the number of cases, changing from 3,064,739 in 2023 to 7,253,599 in 2024 (Table 1). These data represent 83.4% of total cases in 2024, a notable increase compared to 66.7% in 2023 (Table 1 and Figure 2). Argentina also experienced a significant increase, going from 146,876 cases in 2023 to 498,091 in 2024, which increased the proportion from ~3 to ~6%. Conversely, Mexico decreased the number of cases from 277,963 to 73,532, from 2023 to 2024, representing a proportion from 6.0 to 0.8%. Other countries presented a considerable increase in the number of cases, such as Paraguay, from 63,216 to 278,827, and Colombia, from 131,784 to 157,097. In contrast, countries such as Nicaragua and Bolivia presented significant reductions in cases (Table 1). These data indicate that dengue continues to be a major public health problem in Latin America. The significant outbreak of DENV cases in Central and South America signals potential for similar outbreaks not only in Latin America but also in colder regions. A study published in 2024 concluded that climate change has facilitated the spatial spread of West Nile Virus (WNV) in Europe. Additionally, warm and wet days contribute to the movement of mosquitoes into higher latitudes and altitudes. In 2024, Italy and France reported 22 locally transmitted dengue cases, and a locally transmitted chikungunya case was detected in France. Therefore, the increase in DENV cases in some countries in Latin America may be just the tip of the iceberg, foreshadowing what could happen in the coming years regarding the spread of arboviruses worldwide.

The observed temperature anomalies in South America have shown a notable upward trend, increasing by approximately 1 °C over a span of two years, from 0.75 °C in 2022 to 1.75 °C in 2024 (Figure 2b). In 2024, Latin America reported the highest incidence of dengue cases, likely driven by increased vector proliferation resulting from warmer temperatures and heightened precipitation. This trend underscores the direct correlation between rising temperatures, humidity, and frequency of dengue outbreaks. A recent study demonstrated a positive correlation between El Niño events and an increased *Ae. aegypti* larval index in São Paulo State, Brazil. The larval index was notably higher when seasonal rainfall exceeded 153.12 mm and temperatures rose above 23.3 °C [54]. These findings suggest that the combination of climate change and the El Niño event in 2024 likely amplified mosquito proliferation, potentially contributing to the dengue outbreak in Brazil.

Brazil, in particular, saw an approximate tenfold increase in Oropouche virus (OROV) fever cases from 2023 to 2024, underscoring the role of mosquitoes in the spread of arboviruses under favorable climatic conditions. Dengue-related deaths in Latin America highlight significant regional disparities, with Brazil also experiencing the highest number of deaths from 1 January to 5 May 2024, with 3086 cases (Table 2). Argentina, Paraguay, and Peru also reported substantial deaths (343, 100, and 192, respectively), underscoring vulnerabilities in healthcare systems amid expanding outbreaks. Even smaller nations, such as Panama, Uruguay, and Grenada, may reflect underreporting of cases compared to countries with the highest numbers, such as Brazil, Argentina, Paraguay, and Peru (Table 2).

**Figure 3:**
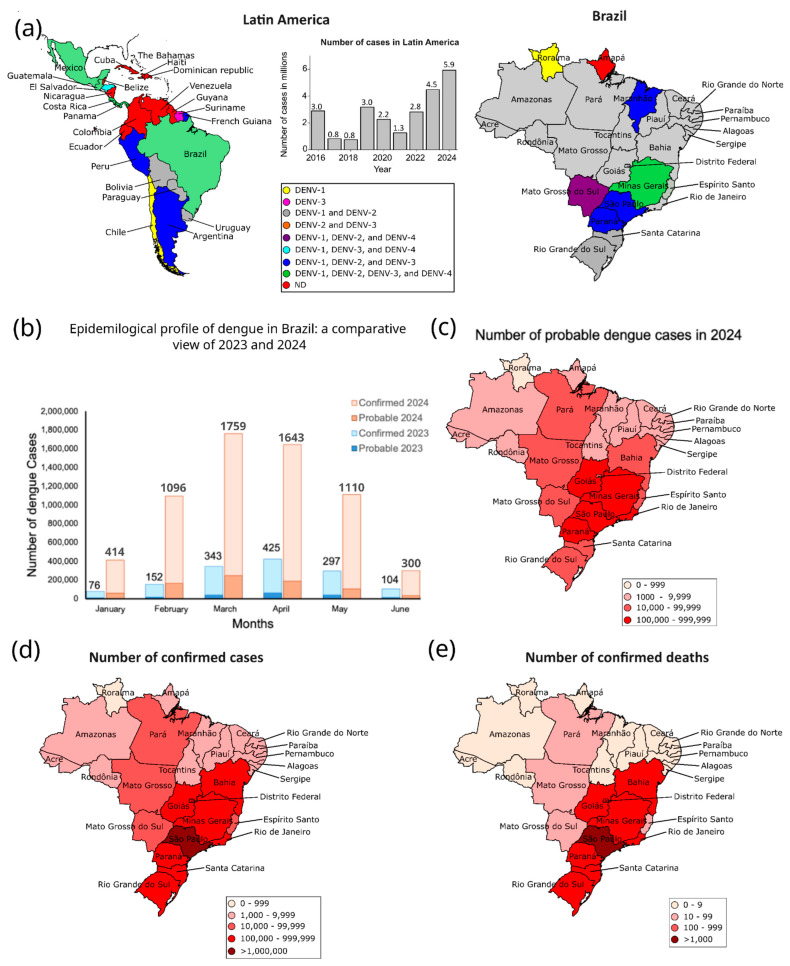
Geographic distribution of serotypes and dengue cases in different regions of the Americas and Brazil, 2024. (**a**) Distribution of the serotypes by country in Latin America from 2016 to November 2024 [57,58]. Latin America comprises 20 countries: Argentina, Bolivia, Brazil, Chile, Colombia, Costa Rica, Cuba, Ecuador, El Salvador, Guatemala, Haiti, Honduras, Mexico, Nicaragua, Panama, Paraguay, Peru, the Dominican Republic, Uruguay, and Venezuela. Twenty-five countries and territories reported the circulation of Dengue serotypes in the Americas. Brazil, Costa Rica, Guatemala, Honduras, Mexico, and Panama reported the simultaneous circulation of DENV-1, DENV-2, DENV-3, and DENV-4 [59,60]. In Brazil, Minas Gerais state reported the simultaneous circulation of DENV-1, DENV-2, DENV-3, and DENV-4 [61]. ND = Not divulged. (**b**) Number of probable (under investigation) and confirmed dengue cases in Brazil from January to June 2023 (blue) and 2024 (orange). Values above each column represent the combined total of cases in thousands [57]. The maps display the number of probable (panel (**c**)) and confirmed (panel (**d**)). Dengue cases across Brazilian states in 2024 [57]. (**e**) Number of deaths caused by dengue in Brazil in 2024. Confirmed DENV cases in Brazil are determined through laboratory testing and clinical–epidemiological criteria. In 2024, 37% of cases were confirmed via laboratory tests, while 63% were based on clinical–epidemiological assessment. The number of confirmed deaths was determined based on laboratory and/or clinical–epidemiological criteria. The number of dengue cases under investigation refers to those that have been officially reported.

In 2023 and 2024, the distribution of DENV serotypes in Latin America varied significantly among countries [55] (Figure 3a). In 2023, most countries, including Antigua and Barbuda, Argentina, Barbados, and Brazil, reported the circulation of DENV-1, DENV-2, and DENV-3, with DENV-4 being less common. By 2024, there was an increase in the diversity of serotypes in many countries, with Brazil, Costa Rica, Guatemala, and El Salvador maintaining the presence of all four serotypes. Argentina and Mexico also reported all four serotypes but Guyana and Trinidad, and Tobago exhibited sporadic presence of some serotypes. Saint Barthelemy and Saint Vincent and the Grenadines reported only one serotype, predominantly DENV-1. The data highlight a notable increase in serotype diversity in many Latin America countries in 2024 compared to 2023 [55]. Since 2021, Dengue cases in Latin America have shown an exponential increase, with 2024 reporting 2.7 times more cases than the previous year (Figure 3a).

Dengue is a notifiable disease in Brazil, meaning that all suspected and/or confirmed cases must be reported to the Epidemiological Surveillance Service of the Municipal Health Department (SMS). Suspected dengue cases can be confirmed through either laboratory criteria or clinical–epidemiological linkage. Patient history, clinical symptoms, and additional medical record information support epidemiological surveillance in the investigation and closure of cases within the official reporting system. Laboratory confirmation is based on serological tests (IgG and/or IGM), detection of NS1 antigen and RT-PCR (Reverse Transcription Polymerase Chain Reaction). The criteria applied are as follows: detection of reactive NS1 protein, positive viral isolation, detectable RT-PCR (within five days of symptom onset), IgM detection by ELISA (from the sixth day onwards), and a ≥4-fold increase in antibody titers in PRNT (plaque reduction neutralization tests) with paired samples (acute and convalescent phases, with at least a 14-day interval). Due to cross-reactivity between the Dengue and Zika viruses, serological tests may yield inconclusive results. When laboratory confirmation is not feasible or results are inconclusive, epidemiological linkage to a confirmed case, supported by spatial distribution analysis, should be used for diagnosis [56].

In March of 2024, the number of probable and confirmed cases of DENV in Brazil was five times higher than 2023, with higher cases and deaths in the southeast and south regions (Figure 3b–e). The cases of DENV may be underestimated due to economic factors and limited diagnostic tests, especially in the north and northeast regions of Brazil. In 2023, Brazil recorded 1.4 million confirmed dengue cases, with an additional 240,462 cases under investigation and 1179 deaths [57]. The fatality rate for dengue cases under investigation is 0.07%, while for severe dengue cases, it rises to 4.8% [57]. On September 26, 2024, Brazil had reported a total of ~5.5 million confirmed dengue cases and 5428 deaths [57]. Additionally, ~1.0 million dengue cases and over 1200 deaths are still under investigation [57]. The fatality rate for dengue cases under investigation is 0.1%, while for severe dengue cases, it rises to 5.5% [57]. The dengue outbreak in Brazil in 2024 occurred between February and May, with more than 300,000 cases under investigation each week. This period corresponds to the late summer and early autumn. The unprecedented rise in dengue cases in Brazil in 2024 is likely due to multiple factors, including conditions that favored mosquito proliferation and an extended period of warm, humid weather. With the onset of autumn and winter, we expect a decrease in dengue cases in Brazil, as cooler temperatures are less favorable to mosquito reproduction. However, continued vigilance and preventive measures remain crucial to controlling the spreading of dengue.

## 3. Dengue Symptoms and Determinants for Recurrence and Disease Severity

**Figure 4:**
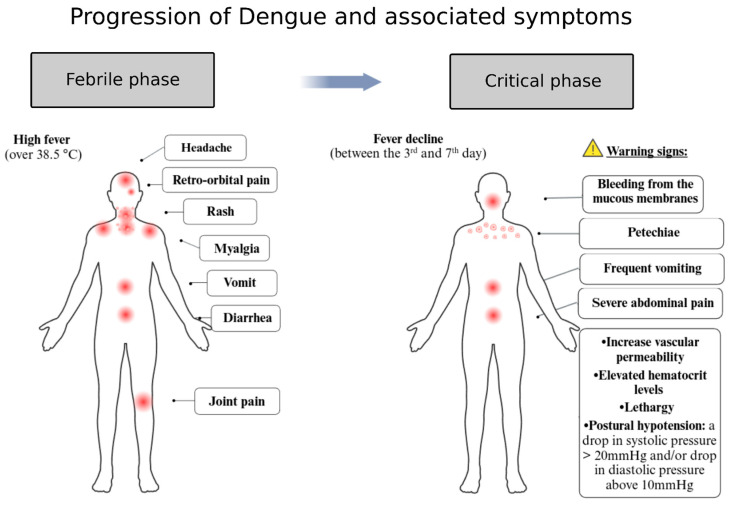
Development and evolution of Dengue symptoms in humans. Dengue progresses through three distinct clinical phases. (1) Febrile phase, which begins after the incubation period and is characterized by classic symptoms such as high fever, rash, myalgia, retro-orbital pain, nausea, and diarrhea (depicted on the left side of the figure). (2) Critical phase, which occurs as the fever starts to subside. During this period, symptoms may worsen, indicating a potential progression to a more severe condition. Symptoms include plasma leakage through capillaries, hemorrhages, shock, and liver or kidney impairment (depicted on the right side of the figure). (3) Recovery phase, where the extravasated fluid is reabsorbed, and gastrointestinal symptoms gradually reduce. This image was created based on data from references [85,86] and designed with BioRender.

Patients infected with Dengue may be asymptomatic or present symptoms ranging from mild to severe, potentially leading to fatal outcomes [62,63]. Dengue is an acute, systemic, dynamic, debilitating, and self-limiting febrile illness. While most recover, some progress to severe forms or death, often preventable with quality healthcare [64]. Symptoms such as sudden fever (39–40 °C) with headache, prostration, muscle/joint pain, or retro-orbital pain warrant immediate medical attention [64]. After the febrile phase (days 3–7), warning signs like severe abdominal pain, persistent vomiting, fluid accumulation (ascites, pleural or pericardial effusion), postural hypotension, lethargy, hepatomegaly (>2 cm), mucosal bleeding, or increased hematocrit may indicate plasma leakage or hemorrhaging [64] (Figure 4). The disease may still progress during recovery, with severe plasma leakage, hemorrhages, or organ failure leading to death [64]. Children often initially show gastrointestinal symptoms like abdominal pain, diarrhea, and vomiting, progressing to classic dengue symptoms. Warning signs in children and infants are subtler, heightening the risk of severe illness. Vulnerable groups include pregnant women, infants, young children, individuals over 65, and those with preexisting conditions [64]. Even mild cases cause significant weakness, impairing daily activities and work performance, with societal financial repercussions. Some patients report nausea, appetite loss, and taste changes, such as a metallic flavor [65].

Although the clinical manifestations of dengue are similar across age groups with symptoms such as fever and headache, a Brazilian study found notable age-related differences in symptom prevalence. Specifically, only 69% of children aged 6–10 and 56% of those aged 1–5 reported experiencing myalgia, compared to over 80% of individuals aged 11 and above. Similarly, arthralgia was observed more frequently in older individuals than in children under 10 years [66]. Interestingly, children under 10 years have an increased risk of hospitalization [67]. However, mortality rates are significantly higher in patients aged 15 years and older, particularly among those over 80 years of age [68].

Common symptoms across all Dengue serotypes include abdominal pain, drowsiness, and occasionally bleeding mucous membranes [69]. However, the presence and severity of other symptoms can vary depending on the serotype. While most primary DENV infections are asymptomatic, they contribute to the infection’s transmission and epidemiological burden [70,71]. In Nicaragua, from 2004 to 2022, DENV-3 was more likely to cause symptomatic and severe infections compared to DENV-1 and DENV-2. Symptomatic infections occurred in 23.2% of DENV-1, 20.1% of DENV-2, and 36.1% of DENV-3 cases, with severe disease observed in 2.5% of DENV-1, 1.4% of DENV-2, and 7.4% of DENV-3 cases, highlighting the variability of clinical outcomes across serotypes [72]. In Vitória, Brazil (2009–2013), DENV-1 accounted for 77.3% of cases, while DENV-2, DENV-3, and DENV-4 contributed 6.4%, 0.2%, and 16.1%, respectively. Among these, 6.6% progressed to severe Dengue, with DENV-2 being strongly associated with severe outcomes [73]. The relationship between DENV serotype and disease severity is complex, influenced by factors such as age, genetic variability, and immune response [74]. Elderly patients are more likely to experience severe dengue due to comorbidities and infections with specific serotypes [75,76], while adults are at higher risk for dengue hemorrhagic fever, and children are particularly vulnerable to severe plasma leakage and shock [77]. These severe manifestations are linked to increased expression of chemokines and pro-inflammatory cytokines (CCL-2, CCL-5, TNF-α, IFN-γ, and IL-6), which act on endothelial cells to promote plasma extravasation and release of pro-inflammatory factors [74,78].

Another critical aspect is that secondary dengue infections are generally more severe than primary infections. This increased severity is believed to occur because a primary infection induces permanent immunity against the initial serotype and short-term immunological memory to other serotypes. During a secondary infection by a different serotype, pre-existing antibodies form complexes with the virus that are then captured by receptors on immune cells, leading to internalization and replication of the virus within host cells [62,79]. Recent studies have also indicated that a primary infection with Zika virus followed by a subsequent Dengue virus infection poses a significant risk for developing severe dengue fever, comparable to the severity observed in heterologous primary and secondary Dengue infections [80].

**Figure 5:**
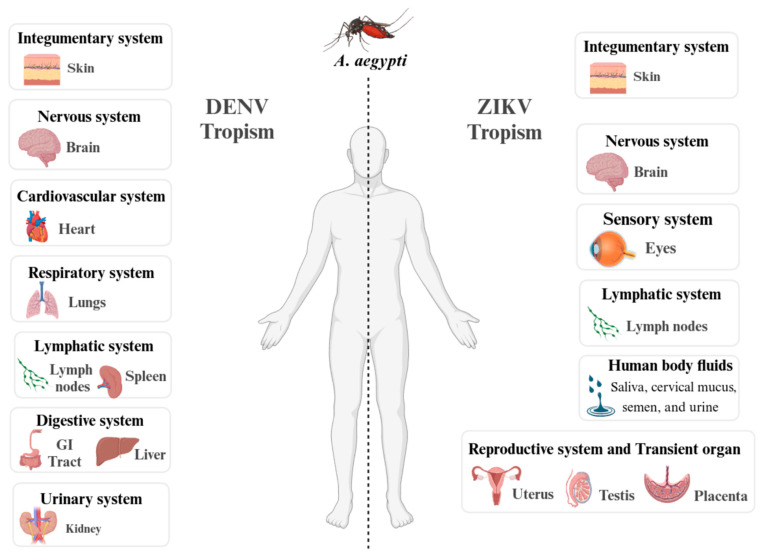
Tissue tropism of Dengue (DENV) and Zika (ZIKV) viruses detected in humans. The organs and body fluids where Dengue virus (DENV, left side) and Zika virus (ZIKV, right side) have been detected in humans bitten by *Ae. aegypti* mosquitoes carrying these viruses. The figure is based on data obtained from references [87,88,89,90] and designed with BioRender.

Dengue and Zika are related viruses transmitted by the same species of mosquitoes. While Dengue has been well-documented and has affected people worldwide for many years, Zika has only recently caused outbreaks. Despite the similarities between viruses, the tropism of the viruses is different (Figure 5). Complication due to Zika virus infection during pregnancy may cause microcephaly and other congenital malformations in the infant, referred to as congenital Zika syndrome [81]. Another neglected arbovirus, with symptoms similar to dengue are Chikungunya and Oropouche Fever (OF) [19,82]. The Oropouche virus (OROV) is one of the most common *orthobunyaviruses* and it has in recent months spread to several countries in Central and South America, alerting the Pan American Health Organization (PAHO, WHO) on 2 February 2024 [83,84].

## 4. Clinical Management and Therapeutic Intervention in Dengue Infection in Brazil

The severity of Dengue infection plays a critical role in determining the appropriate treatment (Table 3). The Ministry of Health (MS) in Brazil, responsible for promoting public health, emphasizes the importance of classifying patients according to the severity of their condition, as outlined in the *National Dengue Control Guidelines*. This classification system, detailed in the *National Guidelines for the Prevention and Control of Dengue Epidemics* [91], ensures that patients receive proper care, reducing mortality and improving outcomes. By following these guidelines, healthcare providers can manage dengue effectively, prioritize resources, and intervene in a timely manner. Adhering to this structured approach is essential for controlling the impact of Dengue and other arboviruses in the Americas [92,93]. The classification system consists of the following groups.

Group A. For patients with classic dengue symptoms. These patients are treated at Primary Health Care Units (Urgent Care) and are advised to undergo home treatment with oral hydration. If symptomatic, analgesics such as Dipyrone and Paracetamol may be recommended. Salicylates, NSAIDs, and corticosteroids are avoided due to the increased risk of gastrointestinal bleeding.

Group B. For patients exhibiting two or more clinical signs of the acute phase, plus spontaneous bleeding (e.g., petechiae, gingival bleeding, and ecchymosis). These patients are attended at a Secondary Health Care Unit with observation beds and are hospitalized for at least 12 h for oral or intravenous hydration and a complete blood count to monitor hematocrit levels.

Group C. When the fever subsides (days 3–7), patients may show signs of worsening disease, such as lethargy, severe abdominal pain, postural hypotension, vomiting, mucosal bleeding, increased hematocrit, and decreased platelet levels. These patients should be transferred to a Tertiary Health Care Unit (Reference Hospital), where they will receive rigorous intravenous hydration (physiological saline or Ringer Lactate) and hourly clinical reassessment, including hematocrit evaluation after 2 h.

Group D. For patients exhibiting shock symptoms such as low blood pressure (BP < 20 mm Hg), cyanosis, rapid pulse, and slow capillary refill. Immediate intravenous hydration with isotonic solutions is necessary at any healthcare facility, followed by transfer to a Tertiary Health Care Unit with ICU beds. Clinical reassessment occurs every 15–30 min, with hematocrit reassessment after 2 h. If treatment is ineffective, further tests and assessments are required, including monitoring of renal function, platelet levels, liver enzymes, and imaging tests. Depending on the condition, analgesics, antipyretics, synthetic colloids, albumin, or diuretics may be used for symptom reversal.

To strengthen healthcare systems across the Americas and reduce the burden of diseases and deaths caused by Dengue, Chikungunya, and Zika, the Pan American Health Organization (PAHO) released the 2022 document titled “*Guidelines for the Clinical Diagnosis and Treatment of Dengue, Chikungunya, and Zika*”. This initiative was supported by the World Health Organization (WHO), the International Technical Group of Experts on Arboviral Diseases (GT-Arbovirus International), and specialists in the GRADE methodology. The guidelines were updated using systematic reviews of published research and the expert knowledge of professionals specializing in these arboviruses.

## 5. Measures Adopted in Brazil to Mitigate DENV Cases

Dengue fever exhibits distinct seasonal contamination patterns, with a notable surge in cases and heightened epidemic risks typically observed between October and May of the following year [95]. The precariousness of basic sanitation services, uncontrolled urban expansion, and improper waste disposal are factors that impact the proliferation of the vector and the disease [91,95]. In 2024, global Dengue cases exploded, particularly in the Americas, where 80% of global cases are concentrated. In the first weeks of this year, case notifications increased in eleven countries, including Brazil, which has also recorded the simultaneous circulation of the four Dengue serotypes (Figure 3) [96]. Climate-change-induced alterations have accelerated mosquito breeding and virus dissemination, contributing significantly to this scenario [97]. Effective year-round prevention hinges on intersectoral policies involving community engagement and healthcare professionals as pivotal actors. Strategies include mechanical, biological, and chemical control methods, along with governmental regulations and legal measures [87,91,98].

In Brazil, it is up to each citizen to adopt preventive measures in their homes and surroundings, avoiding: (a) the accumulation of containers that can store standing water and later become breeding grounds for mosquitoes, (b) the accumulation of leaves and impurities in gutters and drains, (c) open water reservoirs, and (d) the accumulation of trash and debris. Moreover, citizens should adopt individual protective measures such as installing protective screens on windows (mechanical control) and using repellents that are registered in the Agência Nacional de Vigilância Sanitária (Anvisa), containing in their formulation any of these compounds: *N*,*N*-DIETIL-3-Methylbenzamide (DEET), Hydroxyethyl Isobutyl piperidine carboxylate (Icaridin or Picaridin), Ethyl butylacetylaminopropionate (EBAAP or IR3535), or oil of the Cymbopogon (Citronella) [99]. Endemic Disease Control (EDC) professionals and Community Health Agents (CHA) complement these efforts by visiting homes in order to educate and guide their residents on mosquito prevention and overseeing preventive measures [91].

Interestingly, female *Ae. aegypti* detect ultraviolet light at the shorter wavelength end of the human-visible spectrum, but are unable to perceive red light at the longer wavelength end. Their dichromatic vision likely limits their color discrimination ability. Additionally, *Ae. aegypti* exhibit eye parameters optimized for low-light conditions, aligning with their preference for activity in dim environments. This adaptation enhances their capacity to detect hosts and navigate effectively during dawn and dusk, when ambient light levels are low [100]. Photocontrol has been proposed as an effective method for managing mosquito populations. Specifically, blue light with a wavelength of approximately 420 nm has been identified as a promising method for controlling populations of *Ae. albopictus* and *Culex pipiens* f. *molestus* [101]. Capture assays with *Ae. aegypti* demonstrated that traps using various LED intensities and color combinations achieved different capture rates, with the tricolored trap capturing 100% of female mosquitoes. Although statistical differences between the experimental groups were not significant, the tricolored trap appears to enhance female capture rates by accommodating variations in mosquito vision. This green technology-based trap shows promise as an effective, safe tool for reducing mosquito populations and potentially curbing the spread of mosquito-borne diseases [102].

Chemical control entails World Health Organization (WHO)-approved insecticides, including an aerosol spray called “fumacê” in Brazil, an insecticide composed of Prallethrin, Imidacloprid, and solvents, which acts on the mosquitoes nervous system, causing their death. Because it is a method that generates environmental impact and can induce increased mosquito resistance, it should be used sparingly [91,103]. As an alternative to the use of chemical insecticides, the MS incorporated the use of the biological larvicide *Bacillus thuringiensis israelensis* (Bti) into the vector control program, which kills the larvae of *Ae. aegypti*, which ingest its endotoxins. Another highly effective strategy used by the MS has been the *Wolbachia* Method, which involves the release of *Ae. aegypti* mosquitoes infected with the *Wolbachia* bacteria. By reproducing with local mosquitoes, new populations of mosquitoes with the bacteria are formed, which in turn, prevents the development of the virus in the mosquito, interrupting arbovirus transmission. The method has been used in some Brazilian cities that have achieved an important reduction in dengue, chikungunya, and zika cases. For instance, cities like Niterói and parts of Rio de Janeiro observed reductions of 69% and 71%, respectively, following *Wolbachia* deployments in *Ae. aegypti* mosquitoes [104,105]. In Yogyakarta, Indonesia, *Wolbachia* reduced dengue incidence by 77.1% [106,107], while in Colombia’s Aburra Valley, it decreased cases by 95–97% during the 2–4 years compared to the 10 years pre-intervention [108]. These outcomes confirm *Wolbachia* as a powerful tool for reducing dengue in high-risk areas. Recently, The *Wolbachia* Bio Factory was inaugurated in Belo Horizonte city, as a result of a partnership between the MS, the Fundação Oswaldo Cruz (Fiocruz), the patent owner World Mosquito Program (WMP), the government of Minas Gerais, and the Belo Horizonte city [109,110]. This will allow expanding the *Wolbachia* method to additional Brazilian cities and could significantly reduce dengue cases nationwide, as it has already shown promising results in a few test locations.

Alternative strategies for controlling mosquito populations include the development of RNAi-based bioinsecticides, which can be used independently or in conjunction with conventional insecticides. A promising low-cost bioinsecticide has been proposed for direct application to breeding water, derived from lysates of recombinant *E. coli* expressing double-stranded RNA. This bioproduct disrupts the chitinous structures of mosquito larvae, which are critical for their survival. Furthermore, this RNAi-based approach has demonstrated an insecticidal adjuvant effect when combined with diflubenzuron, a chitin synthesis inhibitor, enhancing overall efficacy in mosquito control [106].

Legal measures enforce cleanliness norms on vacant or abandoned properties, facilitating access for health agents in inactive or resistant premises [91]. Brazil’s Unified Health System (SUS) incorporated the Qdenga vaccine (Takeda Laboratory) against Dengue into the National Vaccination Schedule in December 2023, as an additional form of disease progression control. The immunizations for the first dose began in February 2024 and covered 521 municipalities across the national territory. Initially, the priority for vaccinations was children and teenagers between the ages of 10 and 14, who will receive two doses of the vaccine over a three-month period [111]. Despite supply challenges, subsequent vaccine shipments are ongoing, with nearly 1 million doses distributed by late May 2024 to 405 municipalities [97,112]. Unfortunately, although the MS has acquired a large quantity of vaccines, they will not be enough to vaccinate the entire population.

In general, combating dengue fever in Brazil requires a multidisciplinary approach that integrates community involvement, robust healthcare strategies, and coordinated efforts across various sectors. Addressing the environmental, social, and health-related determinants of Dengue transmission is essential for reducing the DENV circulation. Despite implementing various control efforts, Brazil has achieved modest progress in reducing Dengue incidence, with mosquito proliferation increasingly driven by climate change. To prevent another outbreak during the upcoming seasonal cycle, expected from December to May, additional proactive strategies are urgently needed.

## 6. Dengue Vaccines

In Brazil, the search for an effective vaccine against dengue has been a public health priority due to the high incidence of the disease in various regions. Currently, two live attenuated tetravalent Dengue vaccines have been licensed [113]. The first is Dengvaxia (CYD-TDV), which was developed by Sanofi Pasteur. It was approved by Anvisa in 2015 and subsequently in several countries [114], it is recommended for individuals aged 9 to 45 who have had previous exposure to the Dengue virus, being administered in three doses [115]. Dengvaxia primarily induces a CD8+ response targeting NS3 and neutralizing antibodies mainly against DENV-4 [116]. This vaccine is a chimeric live attenuated tetravalent vaccine, which means it contains weakened forms of the Dengue virus that stimulate the immune system without causing the disease [117]. It is based on a vaccine strain of the yellow fever virus (YFV) known as 17D. In this technology, the pre-membrane (prM) and envelope (E) genes of YFV were replaced by the corresponding genes of each of the four DENV serotypes, derived from DENV isolates collected in Thailand and Indonesia between 1978 and 1988. Four chimeric YF-DEN viruses were generated and used in the formulation of the tetravalent vaccine, called ChimeriVax™ DENV 1-4 [118,119,120]. The vaccine offers varying levels of protection for each serotype, i.e., 50.3% for DENV-1, 42.3% for DENV-2, 74.0% for DENV-3, and 77.7% for DENV-4 [121]. Studies showed that seronegative individuals vaccinated with Dengvaxia have an increased risk of developing more severe forms of the disease if they become infected after vaccination, leading to more restricted recommendations for its use [122].

Although Dengvaxia marked a significant advancement in dengue prevention, its implementation requires careful evaluation of the risks and benefits, particularly in populations without previous exposure to the virus. The vaccine remains a milestone in dengue prevention but highlights the need for ongoing development of safer and more effective vaccines to protect all at-risk populations.

The second licensed vaccine is TAK-003, also known as Qdenga or Takeda. Developed by Takeda Pharmaceuticals [123,124], it is a live attenuated tetravalent Dengue vaccine based on the structure of serotype 2 (DENV-2) [125,126], with recombinant strains that express surface proteins for the other serotypes (DENV1, DENV3, and DENV4) [127]. Qdenga stimulates a response against all four Dengue serotypes, with a more robust response for DENV-2 [127].

Qdenga is considered a safe vaccine for both seropositive and seronegative individuals, making it appropriate for use in non-endemic regions for dengue and in both adults and children living in dengue-endemic areas such as Asia and Latin America [128]. Countries including Brazil, Argentina, Thailand, Indonesia, and Great Britain, as well as the European Union, have authorized the use of Qdenga [123]. Clinical studies have demonstrated that TAK-003 may reduce the risk of severe disease and hospitalization. However, it does not completely prevent Dengue infection [129,130].

Achieving high vaccination coverage, particularly in endemic regions, remains a key challenge in the deployment of Dengue vaccines. This coverage is critical to curbing viral transmission. The cost of vaccines further complicates widespread adoption, especially among vulnerable populations. While several other Dengue vaccines are in development, including some in advanced clinical stages, much study remains to ensure their safety and effectiveness across diverse populations. Given the elevated risk of severe disease following a first partial DENV infection, the Laboratory of Infectious Diseases (LID) at the National Institute of Allergy and Infectious Diseases (NIAID), U.S., has employed recombinant DNA technologies to produce a tetravalent live-attenuated Dengue vaccine [131]. Among their developments is TV003, which incorporates non-structural proteins from three Dengue serotypes, contrasting with the CYD™ vaccine that lacks non-structural proteins in its formulation. Preliminary clinical trials demonstrated that TV003 provided significant protection following a second dose administered after 6 or 12 months [131]. Research has revealed that vaccination with TV003 activates both CD4+ and CD8+ T cells, with distinct target preferences: CD8+ T cells largely recognize non-structural (NS) proteins, while CD4+ T cells are responsive to the C and NS2A proteins. Notably, NS3 and NS5 antigens elicit responses from both T cell populations, whereas prM and E proteins trigger lower immune responses. It is plausible that the Butantan-DV vaccine, sharing similarities with TV003, could induce a comparable immunological mechanism [132,133,134].

The Butantan-DV vaccine stems from attenuated strains produced by the NIH and has undergone phase 1 clinical trials in the U.S. The Butantan Institute subsequently selected tetravalent formulations, including TV003, for further development and manufacturing. Currently, the vaccine is in the final stage of clinical trials. Phase 2 trials were conducted in healthy Brazilian volunteers aged 18 to 59, both DENV-naive and previously exposed individuals. Randomized participants received the Butantan-DV vaccine, TV003, or a placebo. The immune responses and neutralizing antibody titers elicited by the Butantan-DV vaccine and TV003 showed no significant differences. Pre-exposure to DENV was correlated with higher neutralizing antibody levels, except for DENV-4. Among DENV-naive individuals, 64% developed an immune response against all four serotypes, while 55% of previously exposed participants achieved a response post-vaccination with Butantan-DV [133].

Phase 3 trials initiated in 2016, enrolling 16,235 healthy participants in a double-blind study. Subjects were divided into three age cohorts: 2–6 years, 7–17 years, and 18–59 years, receiving either the Butantan-DV vaccine or a placebo. Follow-up assessments over subsequent years evaluated the vaccine’s safety and efficacy, particularly against serotypes 1 (89.5%) and 2 (69.6%). Notably, serotypes DENV-3 and DENV-4 were not detected in the trials. The efficacy of Butantan-DV was 80.1% in participants aged 2–6, 77.8% in those aged 7–17, and 90.0% in those aged 18–59. Among DENV-naive participants, vaccine efficacy was 79.6%, compared to 89.2% in those previously exposed. A crucial outcome of the trial was the vaccine’s capacity to prevent severe dengue cases and reduce progression to more serious forms of the disease [113,135].

## 7. Diagnosis Test

Different strategies are employed for dengue diagnosis depending on the stage of the disease and the clinical history of the infection [136] (Table 4). Despite the significant advancements in biomolecular approaches and lateral flow rapid tests, simpler techniques such as the tourniquet test (TT) are widely used in regions with a high incidence of cases and in poor areas where more advanced methods are not affordable or laboratories cannot achieve the demand [137,138]. A positive TT (≥10 petechiae per square inch) is usually observed in patients with dengue hemorrhagic fever and severe forms of the disease due to capillary fragility and thrombocytopenia [139]. However, due to its low sensitivity and specificity, the TT alone is not accepted as a diagnostic criterion and must complement other biomolecular, antigenic, and immunogenic assays [140,141].

When deciding which test to use, previous exposure to Flaviviridae viruses must be considered. In primary infections, a longer viraemia and an extended presence of non-structural protein (NS1) in the bloodstream and body fluids are found. IgM typically emerges around the third day of symptom onset, while IgG is absent until the tenth day. In contrast, immunological memory from past infections allows early production of IgG, creating an effective antiviral response that limits the circulating amount of NS1 [46,142,143,144,145]. Reverse-transcriptase polymerase chain reaction (RT-PCR) is considered a gold standard technique by the World Health Organization for confirming the presence of viral particles, known for its high sensitivity and specificity, which can reach up to 100%. This method is capable of differentiating flavivirus species and even the five DENV subtypes [46,146,147]. However, appropriate sample conservation strategies are essential for reliable RT-PCR results. Samples should be stored at 4–8 °C for no more than 24 h to avoid false-negative results due to the fast degradation of viral RNA *in vitro* [148]. Furthermore, the timing of sample collection during the infection course can impact sensitivity. Viral loads tend to decline as the disease progresses, especially after the fifth day of infection, which may affect detection depending on primer choice, sample processing conditions, and type of commercial kits used [149,150].

For NS1 detection, using Enzyme-Linked Immunosorbent Assay (ELISA), samples can be stored at 4–8 °C for 24 h and at −30 °C or −70 °C for 15 days or longer periods [47,148]. NS1 is a conserved glycoprotein produced by flaviviruses and found in serum and body fluids, making it an interesting target for diagnostic tests. However, saliva and urine samples have shown much lower sensitivity and specificity compared to plasma samples [151,152,153,154].

Serological tests are essential for interpreting disease progression and differentiating between primary and secondary infections. These tests can vary significantly depending on infection history and are susceptible to cross-reactions in individuals previously infected by other flaviviruses, such as a secondary Zika virus infection in patients once exposed to Dengue virus [155]. Positive IgM and negative IgG during the first week of infection indicates a recent primary infection, while positive IgM and IgG at a 1.10 ratio reveals a recent secondary infection (sensitivity (Se) = 100% and specificity (Sp) = 97.4%) [156]. Negative IgM and positive IgG results indicate an infection from the past few months [46,48]. Combining two or more tests (e.g., NS1 and IgM or NS1 and IgM/IgG detections) is strongly recommended to improve sensitivity and specificity (up to 100%), thereby providing a more reliable diagnosis.

In addition to the aforementioned techniques, some older methods, though largely replaced by new technological systems for clinical diagnosis, still hold importance for specific investigations. For example, the plaque reduction neutralization test (PRNT) and virus cultivation require specialized laboratories capable of growing the virus in cell tissues, often taking more than a week to obtain results. PRNT provides information about the presence of antibodies in the patient’s serum that can inactivate specific virus species, while virus cultivation allows for the laboratory culture of viruses from different sources, including autopsy tissues [157,158,159].

Overall, accurate dengue fever diagnosis requires a combination of diagnostic strategies tailored to the disease stage and patient history. Advanced biomolecular techniques and rapid tests have improved diagnostic capabilities, but simpler methods like the tourniquet test are still vital in resource-limited settings. Biomolecular assays, such as RT-PCR and NS1 detection, confirm viral presence and differentiate dengue subtypes, though careful management is needed to ensure accuracy. Serological tests distinguish between primary and secondary infections, despite potential cross-reactions with other flaviviruses. Combining multiple diagnostic methods enhances reliability. Older techniques such as plaque reduction neutralization tests and virus cultivation remain important for specific research. An integrated approach using both advanced and traditional methods is crucial for effective dengue management, with ongoing innovation and improved accessibility key to controlling the disease globally. Table 4 presents recommended diagnostic tests for dengue based on the number of days since symptom onset and the time required to obtain results. It is important to note that the sensitivity (Se) and specificity (Sp) of each test can vary depending on the specific protocols, reagents, and equipment employed for analysis.

Following the incorporation of the Qdenga vaccine (developed by Takeda Laboratories) into Brazil’s National Vaccination Schedule by the SUS in December 2023, vaccinated individuals have shown IgM antibodies for the NS1 protein for over 3 months and IgG antibodies for over a year. This renders traditional serological dengue tests ineffective for this group. The only reliable method for detecting the presence of DENV in these individuals will be through the NS1 antigen test during the period of dengue viraemia or/and RT-PCR. In Brazil, suspected dengue cases can be confirmed through laboratory criteria or clinical–epidemiological linkage. The primary diagnostic tests accepted for confirmation include serological tests (IgG and/or IgM antibodies), detection of the NS1 antigen, and RT-PCR (Reverse Transcription Polymerase Chain Reaction) [56].

**Table 4:** Diagnostic tests based on days after symptom onset. Primary infection (PI) refers to a patient experiencing their first Dengue infection, while secondary infection (SI) pertains to a patient who has had a previous Dengue infection. DF stands for dengue fever, DHF for dengue hemorrhagic fever, Se for sensitivity, Sp for specificity, and RDT for rapid diagnostic test.

Diagnostic Test Timeline (Days After Symptom Onset)					
Method			Primary Infection (PI)	Secondary Infection (SI)	Time
Capillary fragility	Tourniquet Test		- from 0 to 7 days Se = 11.9 to 19.1% in DF and 63 to 83% in DHF [140,160] Sp = 86.4 to 88.9% [140,160] - in DF and 60% in DHF [161]		min
Virus or virus product detection	Virus isolation		- from 0 to 4 days Se = 85.3% [162] - from 4 days onwards Se = 65.4% [162]		≥one week
			Se = 91.0% [162]	Se = 77.6% [162]	
	RT-PCR or RT-qPCR		- from 0 to 5 days Se = 90 to 100% [147,148,149,150] Sp = 100% [150] - from 5 days onwards Se = 38 to 100% [150] Sp = 100% [150]		around one day
	NS1 protein detection	ELISA (serum)	- from 0 to 7 days Se = 93.9 to 100% [154] - from 7 to 9 days Se = 85.7 to 93.9% [154]	- from 0 to 3 days Se = 88.6% [154] - from 3 to 5 days Se = 54.1% [154]	around one day
		RDT (serum)	Se = 80.3% [163] Sp = 100% [163]	Se = 55.1% [163] Sp = 100% [163]	min
			- from 0 to 3 days Se = 76.7 to 83.3% [164] - from 4 days onwards Se = 47.6 to 76.2% [164]		
Antibody detection	IgM detection	ELISA (serum)	- from 4 to 7 days Se = 55% [165] - from 7 days onwards Se = 94% [165]	- from 4 to 7 days Se = 47% [165] - from 7 days onwards Se = 78% [165] (SI patients have a lower concentration of IgM than PI)	around one day
	IgG detection	RDT (serum)	- from 0 to 3 days Se = 3.3% [164] Sp = 100% [164] - from 4 days onwards Se = 23.8 to 38.1% [164] Sp = 100% [164]		min
		RDT (serum)	- from 3 to 7 days Se = 31.82 to 40.91% [166] Sp = 95.24 to 100% [166]	- from 3 to 7 days Se = 82.76 to 95.4% [166] Sp = 95.24 to 100% [166]	min

## 8. Oropouche Fever in Latin America: Rising Incidence, Clinical Overlap with Dengue, and Emerging Public Health Challenges

Oropouche fever was first identified in forestry workers in Trinidad in 1955, with its initial detection in Brazil occurring in 1960 [19,167]. The disease is endemic to several Latin American countries, including Brazil, Panama, Argentina, Bolivia, Ecuador, Peru, and Venezuela, with the Amazon region being particularly affected [19,168]. Prior to the emergence of chikungunya and Zika viruses in 2013, Oropouche virus (OROV) was recognized as the second most prevalent arbovirus in Brazil [169]. Between 1 January and 26 September 2024, Brazil reported 8174 cases of Oropouche fever, a sharp increase compared with 831 cases in 2023, including two deaths. This marks the highest number of cases recorded since the 1961 outbreak, when approximately 11,000 cases were reported [167,170]. Other Latin American countries also experienced an increase in cases during this period, including Bolivia (356), Peru (290), Colombia (74), and Cuba (74). Previous studies in the Brazilian states of Acre and Pará identified four cases of newborns with microcephaly; however, study limitations prevent establishing a causal link between OROV infection and neurological malformations [167,170]. Another study conducted in Brazil detected OROV IgM in 6 out of 68 newborns with unexplained microcephaly, with OROV RNA and antigen present in multiple tissues, including the brain, of one deceased infant [171]. Recently, a case of OROV infection in pregnancy that was associated with a non-apparent malformation stillbirth was reported in Brazil as the first case of vertical transmission. The presence of OROV RNA was detected in the cerebrospinal fluid, brain, lungs, liver, umbilical cord, and placenta with signs of infarction [172]. These findings highlight the need for urgent investigation of the potential role of OROV in fetal harm.

OROV, a member of the *Peribunyaviridae* family, is an arbovirus transmitted primarily by mosquitoes and is the causative agent of Oropouche fever [82]. The transmission of OROV is mainly associated with forested regions and proximity water bodies, with the virus being sustained in nature through both urban and sylvatic cycles [173]. The OROV outbreak can be divided into: (1) sylvatic, transmitted by the mosquitoes *Coquillettidia venezuelensis, Ae. serratus,* and, most importantly, *Culicoides paraenses*, through various hosts, such as sloths (*Bradypus tridactylus*), rodents, birds, and non-human primates; (2) urban, transmitted by the mosquitoes *C. paraenses* and *Culex quinquefasciatus* through humans as the amplifying host [19,82,83,174]. The genus *Culicoides* constitutes a global public health concern, acting as a vector for various arboviruses [82]. Furthermore, climate changes may also contribute to expanding the distribution of OROV vectors to other continents [82,175].

Clinical manifestations of OROV infection include high fever, headache, myalgia, arthralgia, nausea, vomiting, chills, and photophobia symptoms that are often indistinguishable from other arboviruses such as Dengue, West Nile, Zika, Chikungunya, and even Influenza, which may contribute to underreporting [175,176,177,178]. The Oropouche fever symptoms usually disappear within two weeks. A minority of patients may develop severe complications, including hemorrhagic manifestations and neurological involvement [179,180] such as meningitis or encephalitis [19,82,181]. Currently, no specific antiviral treatment or vaccine is available for OROV; thus, patient care remains symptomatic and supportive [181]. Prevention efforts are focused on minimizing mosquito exposure through personal protective measures, such as insect repellents, appropriate clothing, and vector control strategies, as described for preventing Dengue infections. The overlap in symptoms caused by different arboviruses, combined with the dengue outbreak in Brazil in 2024, underscores significant challenges for the healthcare system. With limited capacity for RT-qPCR testing across all samples, most cases were confirmed based on clinical presentation, as previously mentioned. This approach can lead to an overestimation of dengue cases, particularly in endemic regions, where new arboviruses may also emerge. Therefore, the development and implementation of rapid diagnostic tests capable of distinguishing between different arboviruses are essential during outbreak scenarios. These tools would provide a clearer picture of the etiological agents in circulation, allowing the identification of new emerging viruses, and thus, generating more accurate public health responses and resource allocation.

## 9. Dengue’s Emergence in Europe: A Changing Epidemiological Landscape

Dengue outbreaks are becoming more frequent in tropical regions, with increasing reports from colder climates, where such events were previously rare. While the majority of dengue cases have historically been associated with international travel [182], there is a growing shift in this pattern. Anthropogenic climate change has facilitated the expansion of invasive mosquito species into regions where they were once unable to persist. Since May 2024, non-native *Aedes* species, including *Ae. aegypti*, *Ae. albopictus*, *Ae. atropalpus*, *Ae. japonicus*, and *Ae. koreicus*, have been reported as well-established in multiple European countries [183]. This poses a concern for the potential adaptation of native European *Aedes* species as vectors of Dengue and other arboviruses. Notably, *Ae. geniculatus*, a species indigenous to Europe [184], has been implicated in the transmission of chikungunya virus. As arboviruses continue to adapt to the local mosquito population [185], there is a growing risk of their permanent establishment in these new environments.

**Figure 6:**
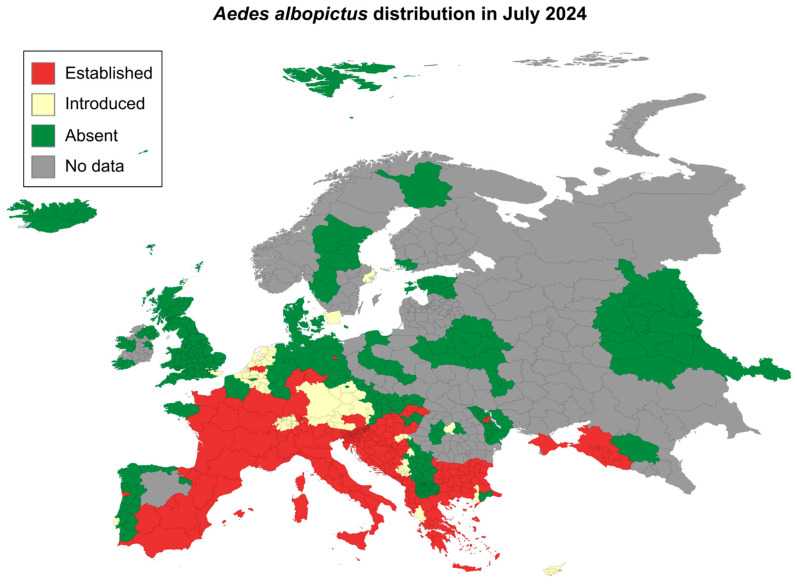
*Aedes albopictus* distribution in Europe. Established populations in the Mediterranean region highlight potential endemic hotspots for Dengue transmission, driven by increasingly favorable climate conditions for the proliferation of *Aedes* species [155].

Among the *Aedes* species, *Ae. aegypti* and *Ae. albopictus* are recognized as primary vectors of DENV. In this regard, *Ae. albopictus* is expected to play a dominant role in the spreading of dengue within Europe (Figure 6) [186]. The northward expansion of *Ae. albopictus*, extending into southern Sweden, raises significant public health concerns. Established populations in the Mediterranean region suggest that these areas may serve as endemic hotspots for Dengue transmission, driven by climate conditions increasingly favorable for *Aedes* spp. proliferation. Another arboviroses of concern in Europe is West Nile virus (WNV). In 2024, the European Centre for Disease Prevention and Control (ECDC) reported 715 locally acquired cases of WNV across 15 European countries. As of September 4, 51 deaths have been attributed to the infection. Around 20% of WNV cases develop into West Nile fever, characterized by symptoms such as fever, headaches, vomiting, and fatigue. In less than 1% of cases, the virus leads to severe neurological complications, including potentially life-threatening swelling of the brain (encephalitis) [187].

**Figure 7:**
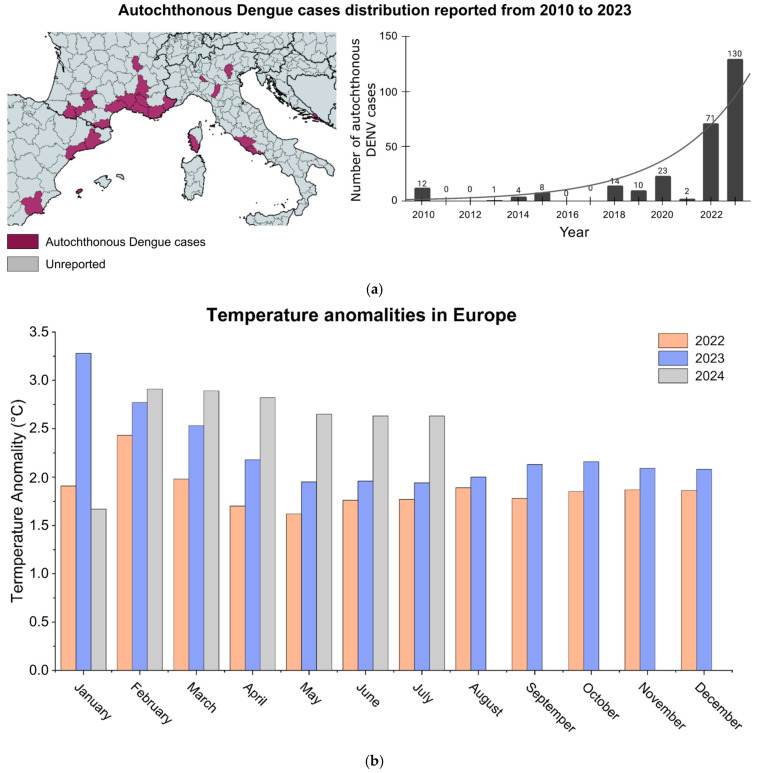
Annual count of total autochthonous dengue cases in Europe. (**a**) Annual count of total dengue autochthonous cases in Europe. The curve represents the exponential regression, showing an increase in total autochthonous cases in Europe as a function of time (in year). (**b**) Temperature anomalies in Europe in the period of 2023 to 2024.

The establishment of non-native *Aedes* mosquitoes in Europe highlights the urgent need for preparedness within healthcare systems and communities. The European Centre for Disease Prevention and Control [186] has documented an upward trend in autochthonous dengue cases across the continent in recent years (Figure 7) [183]. Europe’s climate is undergoing progressive tropicalization, marked by rising temperatures and increased flooding, mirroring environmental conditions in Latin America and other dengue-endemic regions. These climatic changes are closely linked to the introduction and persistence of *Aedes* mosquitoes in flood-prone areas, facilitating their establishment and reproductive success. The growing presence of *Aedes* spp., thus, heightens the risk of Dengue, other arboviruses, and other tropical diseases becoming endemic in Europe.

The exponential profile in DENV native cases is alarming, suggesting a significant increase in cases in Europe (Figure 7a). This underscores the urgent need for European lifestyles and healthcare systems to prepare for managing large numbers of arbovirus cases. With intensifying climate change, proactive measures are crucial to address the presence of *Ae. albopictus* in Europe before dengue and other tropical diseases become endemic in temperate regions. As shown in Latin America, a parallel trend has emerged in Europe, where temperature anomalies similarly increased by approximately 1 °C over two years, rising from 1.75 °C in 2022 to 2.75 °C in 2024 (Figure 7b). The expanding geographical range of mosquitoes in the region highlights the potential risk of future dengue outbreaks driven by favorable climatic shifts. This possibility is further supported by Singh et al., who demonstrated a robust correlation between seasonal temperature rises and annual dengue incidence in India [188].

Therefore, Figure 7 illustrates a concerning rise in autochthonous dengue cases across Europe, highlighting the increasing risk of locally acquired outbreaks. These changes emphasize the urgent need for preparedness, as Europe’s climate is undergoing a ’tropicalization’, which enhances the likelihood of further mosquito proliferation and the potential establishment of other tropical diseases, including Dengue and arboviruses previously undetected in the region. This situation suggests that Europe may not yet be fully equipped to handle the challenges posed by such emerging threats.

## 10. Conclusions

Dengue fever, a prominent arboviral disease transmitted by *Aedes* mosquitoes, is becoming an increasingly critical global public health issue. Factors such as climate change, urbanization, and globalization contribute to the proliferation of mosquito vectors and the cross-border movement of infected individuals. This trend is particularly notable in Latin America, where rising temperatures and increased rainfall foster ideal breeding conditions for mosquitoes. In 2024, Brazil experienced a severe dengue outbreak, with approximately 5.7 million confirmed cases and 5792 deaths, a significant increase from 1.4 million cases and 1179 deaths in 2023 [57]. Additionally, the expansion of *Aedes* mosquito vectors is broadening the virus’s geographical distribution, encroaching upon previously unaffected areas and raising concerns in non-tropical regions about the potential emergence of arboviruses like Dengue. This study underscores the evolving epidemiology of dengue, particularly its appearance in regions traditionally not affected, such as Europe. The establishment of *Ae. albopictus* and other invasive mosquito species in new areas signal a critical shift in DENV transmission dynamics, driven by climate change and vector adaptation [186]. The presence of these vectors in temperate climates highlights significant public health concerns and emphasizes the need for coordinated surveillance, vector control strategies, and preparedness initiatives. Global warming has accelerated the spread of various mosquito species that act as vectors for multiple arboviruses, including Dengue, Zika, Chikungunya, Oropouche, and West Nile viruses. These mosquitoes thrive in warm, humid environments, leading to higher infection rates. The alarming rise in dengue cases in Latin American countries such as Brazil and Argentina in 2024 serves as a warning for Europe and other regions. As a result, while arboviruses have traditionally been endemic to tropical areas, they now represent emerging threats to non-tropical countries. Tackling these challenges will require interdisciplinary approaches and international collaboration to reduce the global spread of Dengue and other arboviruses.

## Figures and Tables

**Table 1:** The total number of dengue cases, including both confirmed cases and cases under investigation, in Latin American countries. This table presents the number of dengue reported cases by country in Latin America in the period of 2023 and until May of 2024. These data were obtained from the Pan American Health Organization (PAHO).

Country	Cases (2023)	Cases (from January to May 2024)
Brazil	3,064,739	7,253,599
Argentina	146,876	498,091
Mexico	277,963	73,532
Paraguay	63,216	278,827
Nicaragua	181,096	17,339
Peru	274,227	242,742
Colombia	131,784	157,097
Bolivia	158,744	36,747
Ecuador	27,838	27,063
Guatemala	72,358	21,991
Chile	ND	148
Uruguay	48	701
Venezuela	4809	ND
French Guiana	2684	14,084
Guyana	27,438	12,929
Suriname	282	95
Nicaragua	181,096	17,339
Costa Rica	30,649	8851
Panama	20,924	6774
Cuba	ND	ND
Honduras	34,050	20,563
El Salvador	5788	2056

ND = Not disclosed.

**Table 2:** Dengue-related deaths by country in Latin America from January to May 2024. This table presents the number of dengue-related deaths in various Latin American countries from January to May 2024.

Country	Deaths Caused by Dengue from January to May 2024	Rate of Death (Death/Cases) (%)
Brazil	3086	0.04
Argentina	343	0.07
Paraguay	100	0.04
Nicaragua	ND	ND
Peru	192	0.08
Colombia	70	0.05
Mexico	26	0.04
Bolivia	14	0.04
Ecuador	31	0.12
Guatemala	10	0.05
Chile	ND	ND
Honduras	10	0.05
El Salvador	ND	ND
Guyana	2	0.02
Panama	12	0.18
Uruguay	2	0.29
Venezuela	ND	ND
French Guiana	ND	ND
Suriname	3	3.16
Costa Rica	ND	ND
Cuba	ND	ND

ND = not disclosed.

**Table 3:** Risk group classification and clinical approaches for dengue treatment. Patients with DENV are classified into distinct severity groups based on clinical signs, hypotension, and bleeding. It guides healthcare professionals with indicators for patient management. Treatment strategies vary according to each group. Group A receives care at Primary Health Care Units with home-based treatments, Group B is observed in Secondary Health Care Units with intensified hydration, Group C undergoes intravenous hydration in Tertiary Health Care Units, and Group D requires immediate intensive care [91].

Group	Signs of Shock	Clinical Approach	Reference
A	ND	- Care provided at Primary Health Care Units (PHC). - Oral hydration and symptomatic treatment with Dipyrone or Paracetamol. - Avoid salicylate-class drugs and non-steroidal anti-inflammatory drugs due to the risk of gastrointestinal bleeding.	[85,87,91,94]
B	Two or more clinical signs of the acute phase, along with spontaneous bleeding (petechiae, gingival bleeding, ecchymosis).	- Care at a Secondary Health Care Unit with observation for at least 12 h. - Oral or intravenous hydration. - Complete monitoring of hematocrit levels.	[85,94]
C	Progression to a more severe clinical condition: lethargy, severe abdominal pain, frequent vomiting, mucosal bleeding, increased hematocrit, decreased platelet count.	- Transfer to a Tertiary Health Care Unit. - Rigorous intravenous hydration with saline solution or Ringer Lactate 1–3 times daily. - Clinical reassessment and hematocrit monitoring every 2 h.	[85,94]
D	Shock: convergent blood pressure (SBP < 90 mmHg), arterial hypotension, cyanosis, rapid pulse, slow capillary refill.	- Immediate care at any health facility with transfer to a Tertiary Health Care Unit (ICU available). - Immediate isotonic intravenous hydration (can be repeated up to three times if needed). - Clinical reassessment every 15–30 min with hematocrit monitoring.	[85,91,94]

ND = Not disclosed.

## Data Availability

Not applicable.
